# Influence of Androgen Receptor in Vascular Cells on Reperfusion following Hindlimb Ischaemia

**DOI:** 10.1371/journal.pone.0154987

**Published:** 2016-05-09

**Authors:** Junxi Wu, Patrick W. F. Hadoke, Kaloyan Takov, Agnieszka Korczak, Martin A. Denvir, Lee B. Smith

**Affiliations:** 1 MRC Centre for Reproductive Health, University of Edinburgh, The Queen's Medical Research Institute, 47 Little France Crescent, Edinburgh, EH16 4TJ, United Kingdom; 2 University/ BHF Centre for Cardiovascular Science, University of Edinburgh, The Queen's Medical Research Institute, 47 Little France Crescent, Edinburgh, EH16 4TJ, United Kingdom; INSERM, FRANCE

## Abstract

**Aims:**

Studies in global androgen receptor knockout (G-ARKO) and orchidectomised mice suggest that androgen accelerates reperfusion of the ischaemic hindlimb by stimulating angiogenesis. This investigation used novel, vascular cell-specific ARKO mice to address the hypothesis that the impaired hindlimb reperfusion in G-ARKO mice was due to loss of AR from cells in the vascular wall.

**Methods and Results:**

Mice with selective deletion of AR (ARKO) from vascular smooth muscle cells (SM-ARKO), endothelial cells (VE-ARKO), or both (SM/VE-ARKO) were compared with wild type (WT) controls. Hindlimb ischaemia was induced in these mice by ligation and removal of the femoral artery. Post-operative reperfusion was reduced in SM-ARKO and SM/VE-ARKO mice. Immunohistochemistry indicated that this was accompanied by a reduced density of smooth muscle actin-positive vessels but no change in the density of isolectin B4-positive vessels in the gastrocnemius muscle. Deletion of AR from the endothelium (VE-ARKO) did not alter post-operative reperfusion or vessel density. In an *ex vivo* (aortic ring culture) model of angiogenesis, AR was not detected in vascular outgrowths and angiogenesis was not altered by vascular ARKO or by exposure to dihydrotestosterone (DHT 10^−10^–10^-7^M; 6 days).

**Conclusion:**

These results suggest that loss of AR from vascular smooth muscle, but not from the endothelium, contributes to impaired reperfusion in the ischaemic hindlimb of G-ARKO. Impaired reperfusion was associated with reduced collateral formation rather than reduced angiogenesis.

## Introduction

Global androgen receptor (AR) knockout (G-ARKO) in mice was recently shown to inhibit reperfusion in the ischaemic hindlimb, a model that involves both angiogenesis (endothelial sprouting and formation of new capillaries) and arteriogenesis (growth of pre-existing small collateral arteries/arterioles; also known as collateral growth) [1]. This impaired reperfusion was linked to reduced angiogenesis since it was associated with lower vascular density in hindlimb muscles [1]. Consistent with this proposal, G-ARKO was also shown to inhibit angiogenesis using *in vivo* (silicon tube implantation) and *ex vivo* (aortic ring culture) models [1]. However, AR is widely expressed and these investigations did not determine whether the influence of AR on angiogenesis was associated with a particular cell population. Furthermore, interpretation of these results was complicated by the systemic effects of global androgen deficiency [2,3].

AR is present in cells of the vascular wall, with expression reported in cultured endothelial (EC) and smooth muscle (SMC) cells [4–6]. Therefore, since ECs have a central role in the formation of new vascular networks, it has been proposed that androgen regulates angiogenesis via direct stimulation of AR in ECs [7]. This suggestion was supported by the report that administration of the non-aromatisable AR agonist dihydrotestosterone (DHT) reversed impaired hindlimb reperfusion in castrated male mice [7]. This effect was attributed to AR-induced promotion of EC angiogenesis, based on the observations that DHT promoted AR-dependent migration and angiogenesis in human umbilical vein endothelial cells (HUVECs) [7]. However, in the *in vivo* experiments described by Sieveking *et al*. [7], it was not possible to determine whether the increased angiogenesis and restored hindlimb reperfusion stimulated by DHT were mediated by AR in the vasculature.

We recently described generation, using the cre-loxP system, of a novel murine model of vascular cell-specific ARKO [5]. This line produces mice with selective deletion of AR from the vascular ECs (VE-ARKO), SMCs (SM-ARKO) or from both ECs and SMCs (SM/VE-ARKO), as well as littermate controls. Unlike G-ARKO, vascular ARKO mice have normal testicular development and normal circulating testosterone levels, thus avoiding the systemic side-effects of global androgen deficiency, such as low circulating androgen/oestrogen, altered lipid profile and impaired muscle development [2,3,5]. Here we used our vascular cell-specific ARKO mice to address the hypothesis that the impaired reperfusion reported in G-ARKO mice [1] is caused by the loss of AR from vascular cells.

## Methods

### Mice

Animal experiments were performed in accordance both with Directive 2010/63/EU of the European Parliament and with the UK Home Office Animal (Scientific Procedures) Act 1986, and licensed under Project Licence PPL 60/4523. All procedures were approved by the University of Edinburgh ethical review committee (Animal welfare and Ethical review body).

Mice with selective deletion of AR from SMC (SM-ARKO), EC (VE-ARKO) or both cell types (SM/VE-ARKO) were established in our laboratory, as previously described [5]. All transgenic mice were of C57BL/6J background. Cell-specific ablation of the *Ar* gene has been thoroughly examined and confirmed in freshly isolated aortic endothelial cells and smooth muscle cells from all of the four genotypes [5]. Routine genotyping of stock mice was carried out by examining the inheritance of Cre recombinase [5]. Briefly, genomic DNA was extracted from a frozen ear clip from each mouse. Primers CGCATAACCAGTGAAACAGCATTGC and CCCTGTGCTCAGACAGAAATGAGA were used for Tie2-cre [8]; and primers CGCATAACCAGTGAAACAGCATTGC and CAGACACCGAAGCTACTCTCCTTCC for SM22-cre [9]. PCR amplification products were resolved using a QiaXcel capillary system (Qiagen, UK). An amplicon of 608 bp indicated inheritance of the Cre Recombinase transgene in EC under control of the Tie2 promoter, whilst an amplicon of 575 bp indicated presence of the Cre Recombinase transgene in SMC under control of the SM22 promoter.

Only male mice (18.2±0.5 weeks old) were used in this study. Mice (3-5/ group) were maintained at 21±2°C, 50% humidity, and a 12 hour light/dark cycle with *ad libitum* access to chow and water.

### Hindlimb ischaemia model and laser Doppler imaging

Unilateral hindlimb ischaemia was introduced by femoral artery ligation, as previously described [10]. Briefly, mice were anesthetized by inhalation of isoflurane (5% for induction and 2–3% for maintenance) with appropriate analgesic cover (buprenorphine; 0.05mg/kg body weight, s.c.). Depth of anaesthesia was indicated by loss of the pedal withdrawal reflex. The femoral artery in the left leg was carefully dissected and ligated distal to the inguinal ligament and proximal to the saphenous/popliteal bifurcation. The ligated artery segment was then excised. Wounds were sutured (6–0 Mersilk) and mice were allowed to recover for 21 days. Hindlimb blood flow in the foot pad was measured with a Moor Infrared Laser Doppler Imager (Moor Instruments, UK) immediately before and after femoral artery ligation as well as 3, 7, 14 and 21 days after surgery. To control the variation of systemic perfusion caused by handling stress and fluctuation of body temperature in individual mice, blood perfusion in the food pad (the mean signal intensity of the selected area) was normalised to perfusion in the tail.

### Histology and immunostaining

For histology and quantitative PCR, mice were culled, by asphyxiation in CO_2,_ 21 days following induction of hindlimb ischaemia. The gastrocnemius muscle was harvested both from ischaemic and control legs. Each muscle was cut into two pieces. One half was frozen at -80°C and the other half fixed in 10% neutral buffered formalin for 24 hours and embedded in paraffin. Cross-sections from the middle of the muscle were used for haematoxylin and eosin staining. Total vessels (Isolectin B4 (IB4), Life Technologies; or anti-CD31 antibody, Abcam) and mature arteries/veins (anti-smooth muscle alpha-actin antibody (SMA), Sigma) were identified by immunostaining on serial sections. To quantify the vessel density, a whole cross-section of gastrocnemius muscle was tile-scanned at x200 magnification (Slide scanner Axio Scan.Z1, Zeiss). The effective muscle areas subject to quantitative analysis were 14.0±0.6 mm^2^ for each mouse. The muscle area and vessel density were quantified using Fiji and Image-pro plus 7.0 software (MediaCybernetics, UK). The presence of AR in the vasculature and skeletal muscle was identified by immunostaining using anti-AR antibody (Santa Cruz, USA), and imaged by confocal microscopy.

Whole mount immuno-staining was used for samples from the aortic ring assay. Each collagen gel with the aortic ring was fixed *in situ* by adding ice-cold zinc formalin fixative (100xl: Sigma) for 30 minutes. The sample was permeabilised with phosphate-buffered saline (PBS) containing 0.25% (vol/vol) Triton X-100 (Sigma) for 2x15 minutes. The primary antibodies (for AR and SMA) were added directly into the 96-well plate and incubated overnight at 4°C. Visualization was achieved by incubation with secondary antibodies conjugated with appropriate fluorescent dyes for 1 hour at room temperature and counter-stained with DAPI. The whole piece of collagen gel was then carefully mobilised and mounted on a glass side with Mountant PermaFluor (Thermo Scientific). A cover slip placed on top of the gel with very gentle pressure. Slides were stored at 4°C before imaging.

### Aortic ring assay

*Ex vivo* angiogenesis was tested using the aortic ring assay, as previously described [11]. Briefly, mice were culled by asphyxiation in CO_2_ and the thoracic aorta was isolated. The descending thoracic aorta from all four genotypes were cut into 15 rings and each ring was embedded in 100μl of collagen gel (1% collagen in Opti-MEM) in a 96-well plate. 200μl of Opti-MEM (Gibco, cat. no. 51985–026) containing penicillin-streptomycin and 1% fetal bovine serum (FBS) was added onto the gel. Culture media were changed every 3 days. Dihydrotestosterone (DHT, Sigma, cat. No. A8380) was dissolved in ethanol and diluted to the designated concentration using culture medium. The final concentration of ethanol was less than 0.01% vol/vol. Exposures were performed in triplicate. The number of tube-like structures in each well was manually counted on day 6.

### Statistics

Data were expressed as mean ± standard error of the mean (SEM) where n refers to the number of mice. Data between two groups were analysed using unpaired Student’s t-test. Data from multiple groups were analysed using one-way or two-way ANOVA with Tukey’s post hoc test, as appropriate. Statistical analyses were performed using GraphPad Prism v6.04. Differences were considered significant when p<0.05.

## Results

### Selective deletion of smooth muscle AR impairs hindlimb reperfusion following femoral artery ligation

**Fig 1:**
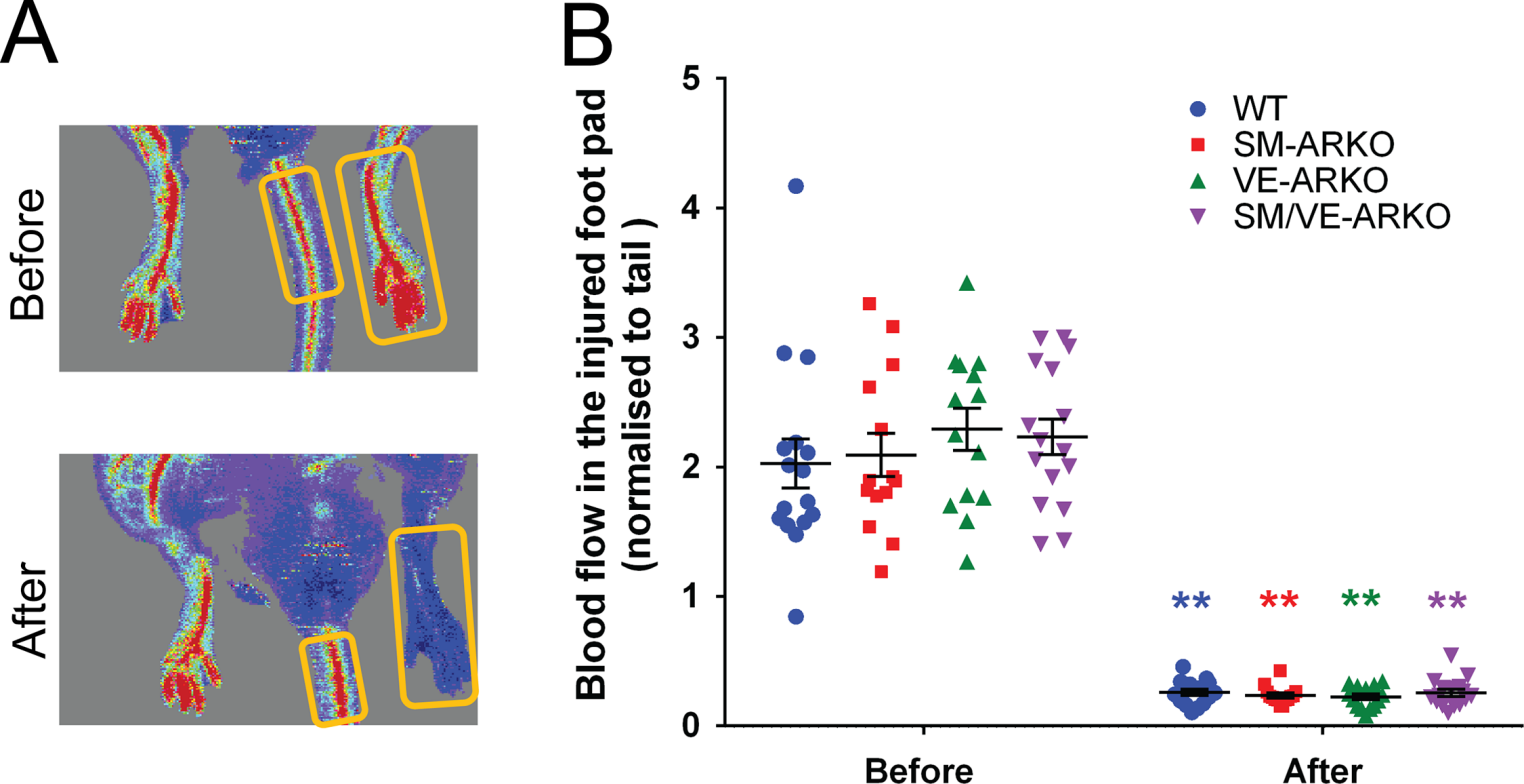
Impact of vascular AR deletion on pre- and post-operative hindlimb perfusion. (A) Laser Doppler was used to monitor perfusion of the foot pad immediately before and after femoral artery ligation, which was quantified by software and normalised to tail. (Areas of interest are indicated by yellow boxes. The grey area is excluded by software automatically.) (B) Perfusion in the un-operated hindlimb was unaffected by selective deletion of vascular AR. Femoral artery removal produced a dramatic reduction in hindlimb perfusion that was similar in wild type and ARKO mice. ** p<0.01 compared with corresponding pre-operative measurements (n = 14–16).

To investigate the role of vascular AR on *in vivo* recovery of blood perfusion in the ischaemic hindlimb, we employed a mouse model of femoral artery ligation. Baseline blood flow (pre-surgery) in the mouse hindlimb was not altered by selective deletion of vascular AR (Fig 1), suggesting that vascular ARKO does not alter normal vascular development in the hindlimb. Femoral artery ligation induced a consistent rapid reduction of blood flow in the foot in all genotypes (Fig 1).

**Fig 2:**
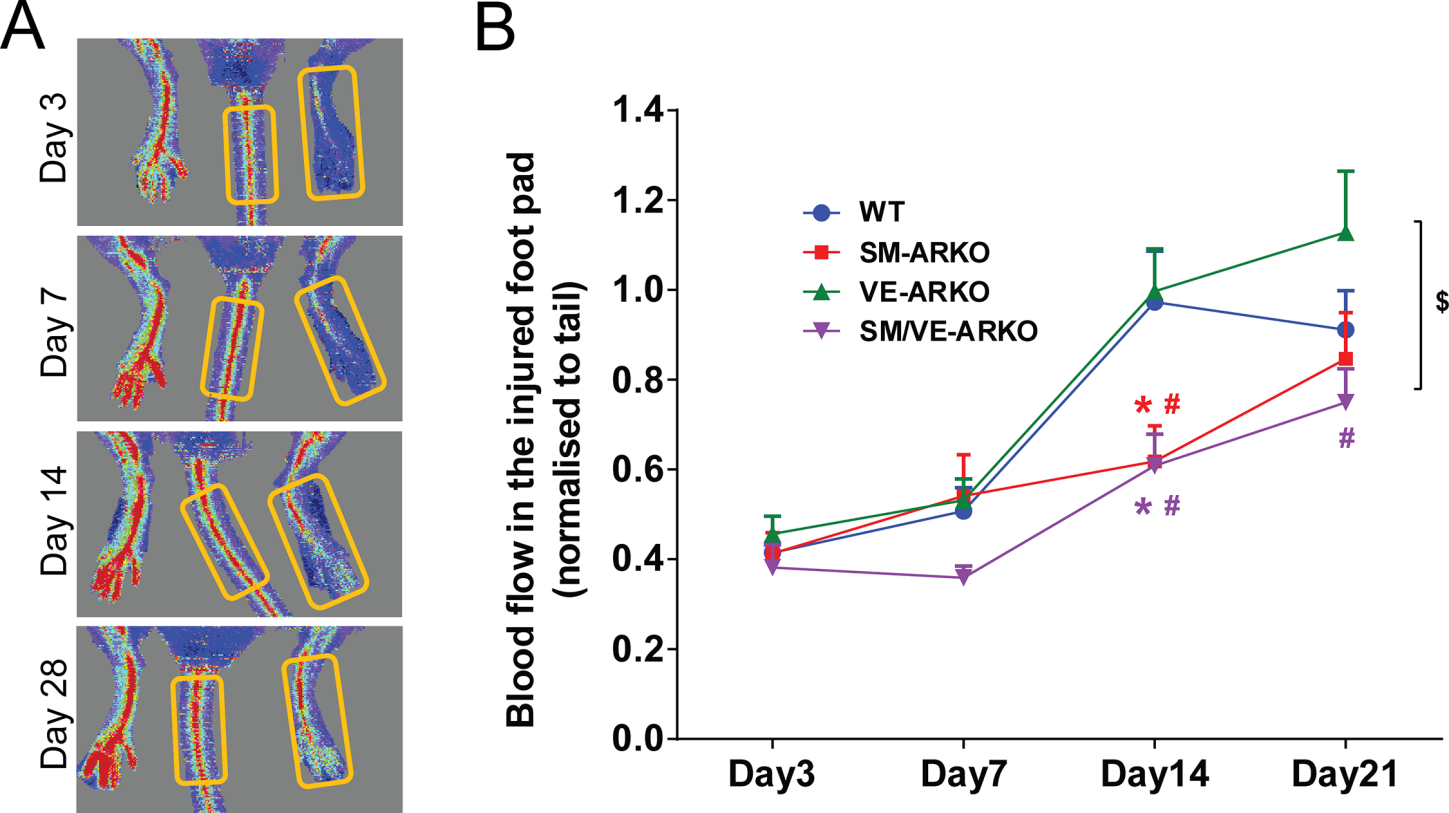
Deletion of AR from smooth muscle, but not from endothelium, reduced post-operative reperfusion of the ischaemic hindlimb. (A) Laser Doppler imaging demonstrated partial restoration of hindlimb perfusion over 21 days following femoral artery removal. The blood flow in the foot pad was quantified and normalised to flow in the tail. (Areas of interest are indicated by yellow boxes. The grey area is excluded by software automatically.) (B) Deletion of AR from smooth muscle impaired this recovery, whereas deletion of AR from endothelial cells did not. $ p<0.01 difference among genotypes; * p<0.01 versus WT; # p<0.01 versus VE-ARKO. Data were analysed by two way ANOVA plus Tukey post hoc test (n = 14–16).

In WT mice femoral artery removal was followed by a gradual recovery of perfusion in the foot during the 1^st^ week following surgery. Recovery accelerated in the 2^nd^ week, and stabilised during the 3^rd^ week (Fig 2). A similar pattern was seen in VE-ARKO mice (Fig 2). In contrast, both SM-ARKO and SM/VE-ARKO demonstrated a blunted recovery especially in the 2^nd^ week following surgery. Consequently, both SM-ARKO and SM/VE-ARKO showed impaired reperfusion at day 14, which remained evident in the SM/VE-ARKO at day 21 (Fig 2; two-way ANOVA, p<0.01).

### The impact of vascular ARKO on post-ischaemic revascularization in the hindlimb

**Fig 3:**
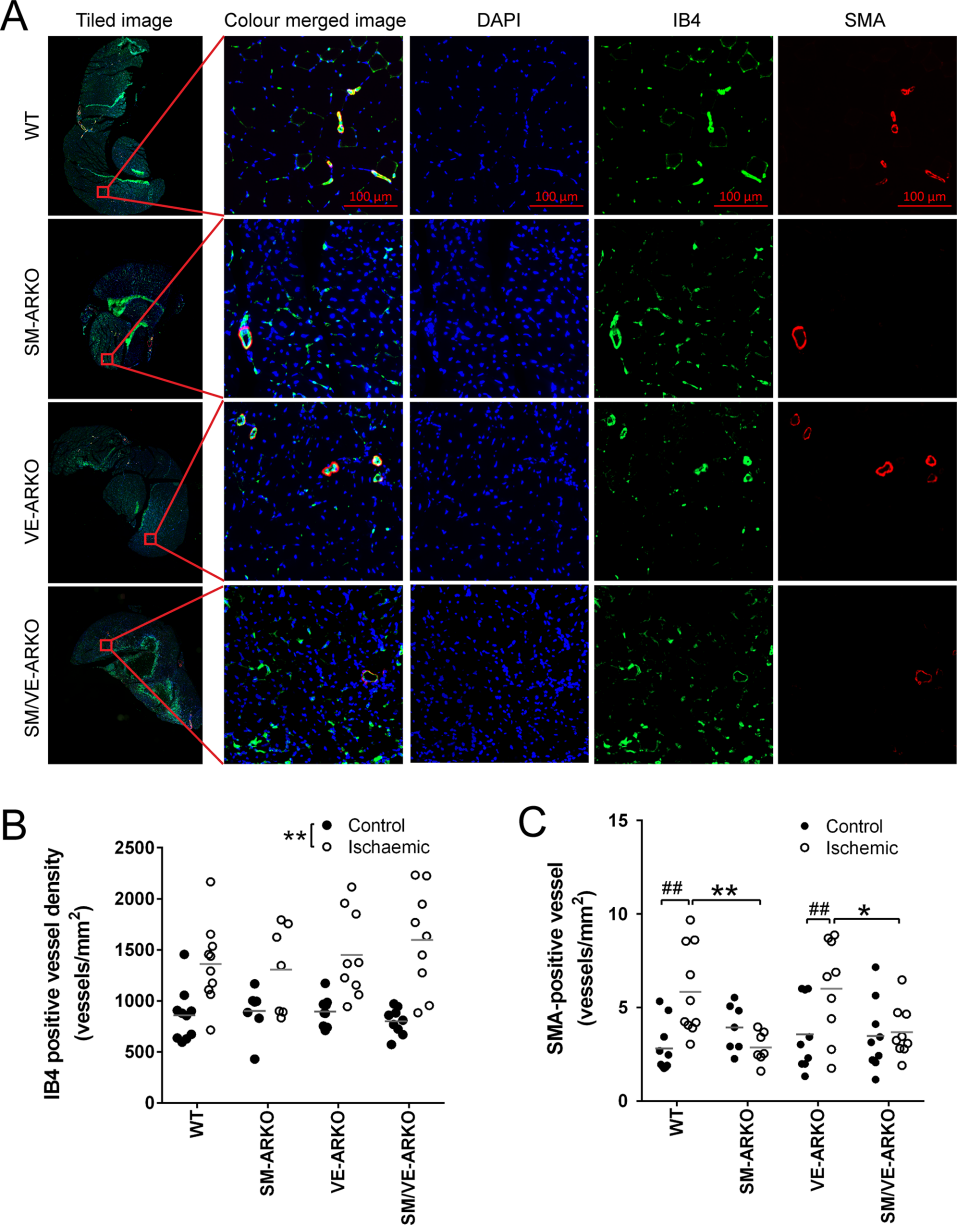
AR deletion from vascular smooth muscle, but not from endothelial cells, altered vascular density in ischaemic gastrocnemius. (A) Detection of endothelial cells (IB4-positive) and smooth muscle cells (SMA-positive) in vascular structures was achieved using immunohistochemistry on cross-sections of gastrocnemius muscle. High resolution tile-scanning (@ x200) using slide scanner Axio Scan.Z1 (Zeiss) allowed quantification of vascular phenotype and density of the whole muscle section. (B) Quantitation of vessel density indicated that deletion of AR from neither vascular smooth muscle nor endothelium altered the rise in density of IB4-positive vessels in ischaemic hindlimb. **, p<0.01 by two way ANOVA. (C) Deletion of AR from vascular smooth muscle, but not endothelium, prevented the ischaemia-induced increase in SMA-positive vessels. * p<0.05; ** p<0.01; by two way ANOVA plus Tukey post hoc test. ## P<0.01, by Student’s t-test (n = 7–10).

To determine whether the altered reperfusion was a consequence of increased angiogenesis or arteriogenesis in the ischaemic tissue, we examined vascular density in the ischaemic gastrocnemius muscle. In gastrocnemius muscles isolated 21 days following surgery, immunostaining by IB4 identified structures (arteries, veins, capillaries) with an EC layer (Fig 3A). Immunostaining against SMA identified medial smooth muscle in mature arteries and veins (Fig 3A).

The density of IB4-positive vessels in gastrocnemius muscle from the contralateral uninjured (Control) hindlimb was similar in wild type and vascular ARKO mice (Fig 3B). Hindlimb ischaemia induced a similar increase in IB4 positive vessel density in all groups (Fig 3B).

The density of SMA-positive vessels in gastrocnemius muscle from the contralateral uninjured (Control) hindlimb was also similar in wild type and vascular ARKO mice (Fig 3C). Induction of ischaemia increased the density of SMA-positive vessels in the gastrocnemius of WT mice (Fig 3C). This increase was not altered in VE-ARKO mice but was abolished by SM-ARKO or SM/VE-ARKO (Fig 3C).

Induction of ischaemia significantly up-regulated mRNA expression levels of several ischaemia/angiogenesis-related genes (*Pdgfrb*, *Pecam1*, *Cdh5*, *Vwf*, *Hif1a*, *and Thbs1*), but did not alter expression of *Vegfa*, in gastrocnemius muscles 21 days following surgery. Expression of these genes was not altered by vascular ARKO (S1 Fig).

### Immunohistologial detection of AR in vasculature of gastrocnemius

**Fig 4:**
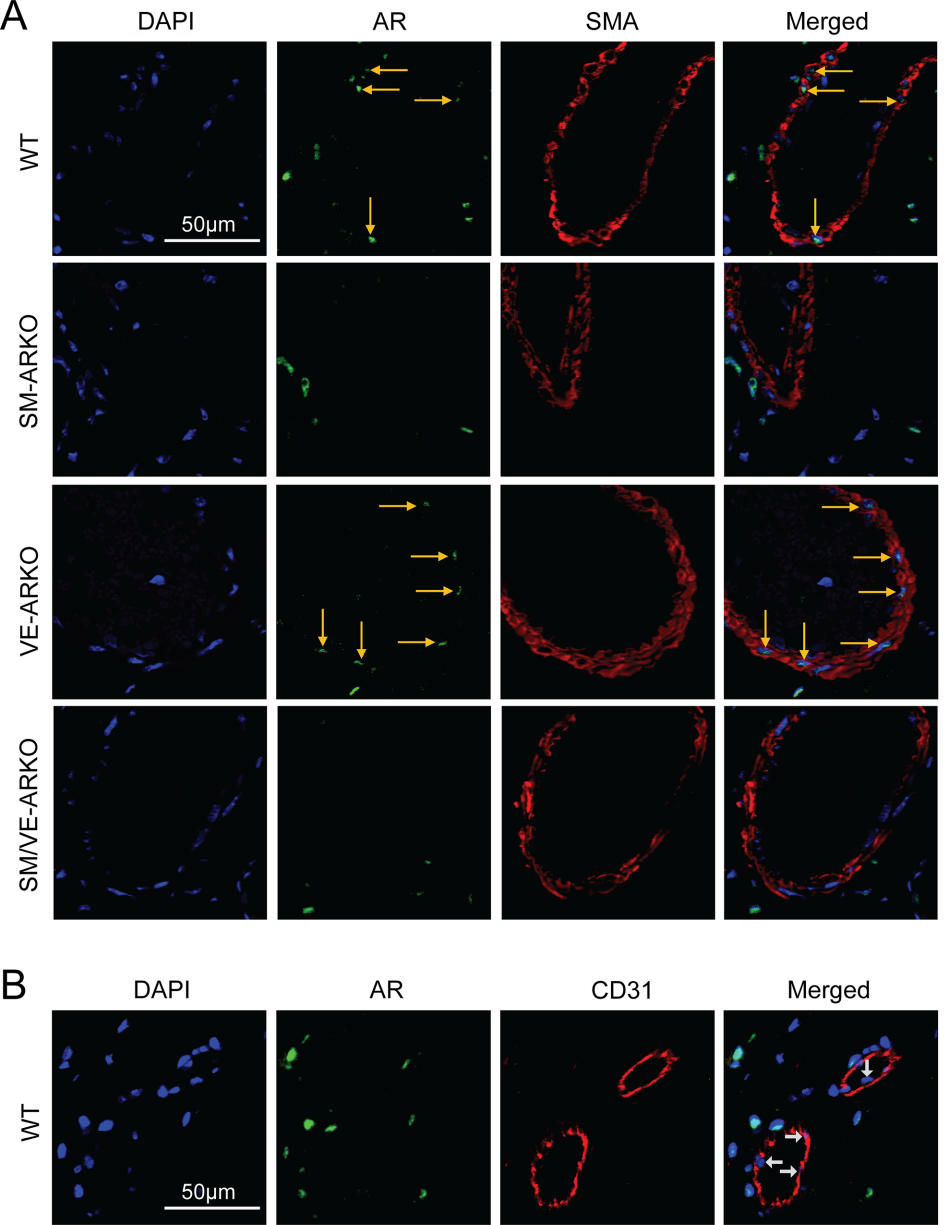
Localisation of vascular ARKO in murine ischaemic gastrocnemius. (A) AR was detected in the vascular smooth muscle (AR = green, SMA = red, DAPI = blue) in the ischaemic gastrocnemius muscle at day 21 from WT and VE-ARKO, but not SM-ARKO and SM/VEARKO. No positive staining of AR could be identified on the luminal (endothelial) side of the vessel in any genotypes. (Yellow arrows indicate AR-positive nuclei) (B) Double staining of AR and CD31 in WT confirmed that AR was not expressed in vascular endothelium in the ischaemic hindlimb. (White arrows indicate nuclei of endothelial cells.) Some AR positive cells were also found in the perivascular connective tissue.

To establish the reason for the cell-specific response to ARKO in hindlimb ischaemia, we examined AR expression in the ischaemic gastrocnemius muscle. AR was identified in SMCs and some perivascular cells in ischaemic gastrocnemius muscle from WT and VE-ARKO, but not from SM-ARKO and SM/VE-ARKO (Fig 4A). AR was not observed on the luminal side of vessels in any of the genotypes (Fig 4A), indicating lack of AR expression in vascular endothelium *in vivo*. This was confirmed by double staining against AR and CD31 in ischaemic WT gastrocnemius muscle (Fig 4B). Vascular ARKO had little impact on the AR expression in skeletal muscle (S2 Fig) and did not alter the level of muscle injury in the ischaemic hindlimb by day 21 (S3 Fig).

### Androgen action on *ex vivo* angiogenesis

**Fig 5:**
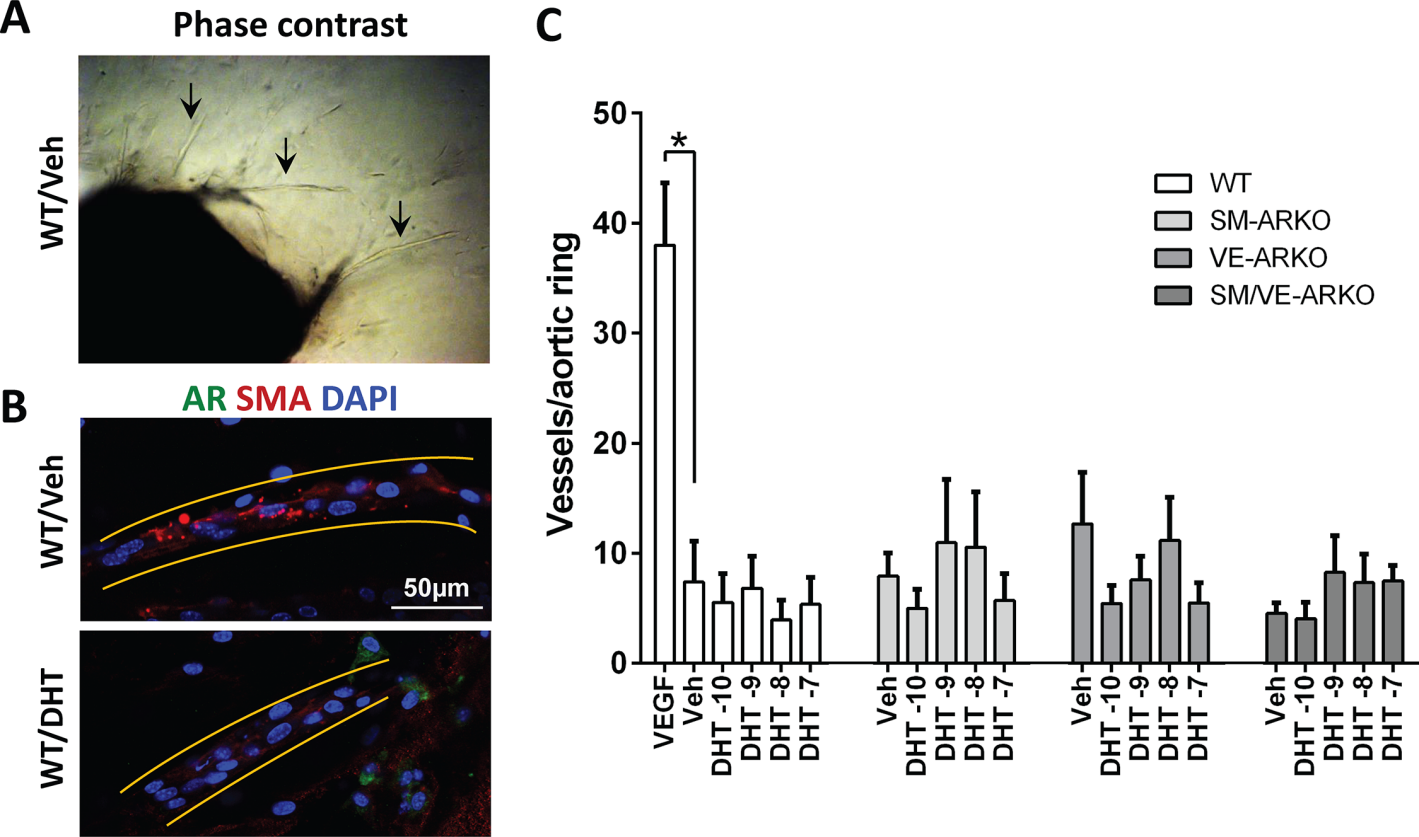
Androgen signalling did not regulate *ex vivo* angiogenesis in the aortic ring assay. Descending thoracic aorta were from all four genotypes were freshly dissected, cut into aortic rings and embedded in collagen gel. Endothelial cell sprouting and tube-like structure formation was stimulated by culture medium with 1% FBS. Tube-like structures were counted manually at day 6. (A) Representative phase-contrast image of tube-like structure (arrows) growing from a WT aortic ring. (B) Representative images of AR expression in tube-like structure from WT aortic rings with/without DHT treatment. Yellow lines highlight a tube-like structure. (C) Neither DHT nor vascular ARKO significantly altered angiogenesis in the aortic ring assay (By two way ANOVA). VEGF (5ng/ml) treatment in WT rings served as positive control for maximum angiogenesis (*, p<0.01 by Student's t-test). Exposures were performed in triplicates, and repeated 6 times, with the exception of the VEGF group, which was repeated 4 times.

To clarify the net contribution of vascular AR in regulation of angiogenesis without the confounder of arteriogenesis, we employed an *ex vivo* aortic ring assay of angiogenesis. FBS (1% in Opti-MEM) stimulated EC sprouting and formation of tube-like structures in aortic rings from WT mice (Fig 5A). Immunohistochemistry demonstrated AR expression was only detectable in some perivascular cells but not in the tubule structure (Fig 5B). Vascular cell-specific ARKO did not alter tube-like structure formation (Fig 5C). Exposure to DHT did not promote tube-like structure formation in aortas from WT or vascular ARKO mice (Fig 5C), despite inducing AR expression in some perivascular cells (Fig 5B).

## Discussion

Recent evidence suggests androgen improves reperfusion in ischaemic disease, although the precise mechanism remains unclear [1,7]. This study addressed the hypothesis that direct activation of vascular AR regulates post-ischaemic revascularization and reperfusion. By applying a combination of *in vivo* and *ex vivo* approaches to mice with vascular cell selective ARKO, we were able to show that deletion of AR from SMC suppressed post-ischaemic reperfusion, due to impaired arteriogenesis. Deletion of AR from the endothelium had little effect on post-ischaemic angiogenesis either *in vivo* or *ex vivo*.

Previous studies demonstrated that androgen depletion (by castration) or G-ARKO impaired reperfusion in hindlimb ischaemia [1,7]. However, due to the important role of AR in skeletal muscle [3,12–16], over 50% G-ARKO ischaemic legs developed autoamputation by day 21, but none in WT [1]. The impaired reperfusion in G-ARKO, therefore, cannot be simply explained by decreased angiogenesis.

The generation of mice with vascular ARKO [5] allowed direct determination of the role of vascular AR in post-ischaemia revascularization, without the systemic complications seen in castrated or G-ARKO mice. Vascular ARKO did not affect AR expression in skeletal muscle and ischaemia-induced muscle injury. Thus, these mice provide a viable model for investigating the role of AR in vascular remodelling.

Direct evidence of AR expression in vascular endothelium *in vivo* is very limited. Our previous data demonstrated that AR was sporadically expressed in mouse aortic endothelium [5]. AR was undetectable (by immunostaining) in the endothelium of smaller vessels, including femoral artery, mesenteric artery and capillaries (data not shown). Consistent with these observations, AR expression was not detected in newly formed endothelial tubules in the aortic ring assay (with or without DHT), nor in the neovasculature in ischaemic hindlimbs. Therefore, it is not surprising that VE-ARKO had no impact on angiogenesis in either *ex vivo* or *in vivo* models.

SMCs (SMA-positive) are known to play a critical role in post-angiogenic vascular remodelling and initiation of blood perfusion [17,18]. Further development of microvessels into conductance vessels or growth of collateral vessels, defined as arteriogenesis, are important mechanisms for reperfusion of ischaemic tissue [19]. Notably, successful post-angiogenesis remodelling can induce a 60% reduction in neo-vascular density to form a mature vascular network [17,20]. Our previous and current studies demonstrated that SMCs constitutively express AR *in vivo* and *in vitro* [5]. Deletion of AR from SMCs suppressed the formation of SMA-positive vessels, suggesting an interruption of arteriogenesis. The persistence of impaired reperfusion suggested that the IB4-postive SMA-negative microvessels are inefficient in conducting blood to remote ischaemic tissue (mouse food pad). Whatever the mechanism, these results suggests that arteriogenesis mediated by AR in SMCs is important for successful hindlimb reperfusion. It is also possible that deletion of AR from SM could reduce hindlimb perfusion by altering vascular contractility. In our previous investigation, however, we demonstrated that arterial contraction in response to adrenoceptor agonists was reduced in SM-ARKO mice, which would predict greater perfusion [5]. Further studies are required to determine the mechanisms underpinning regulation of arteriogenesis by AR in SMCs.

Yoshida *et al*. demonstrated an early rise (day 1) in *Vegfa* expression in G-ARKO ischaemic hindlimb, suggesting a maintained local angiogenic response [1]. Our results extend this observation, indicating that deletion of vascular AR does not impair angiogenesis. However, induction of angiogenesis does not necessarily predict improvement in reperfusion. In order to deliver blood to the ischaemic tissue, neovasculature requires extensive remodelling [17,18,20]. VEGF alone is not sufficient to drive neovascular maturation and arteriogenesis [21]. This was reflected by the failure of clinical trials using VEGF-based therapies aiming to improve post-ischaemic myocardial perfusion or reduce lower limb amputation rate [22]. Additional factors, such as PDGF-B, have a role in recruitment of SMCs/pericytes to stabilise neovasculature and support further arteriogenesis [23–25]. Further investigation is required to clarify whether androgen signalling interacts with PDGF-B signalling in regulation of SMC/pericyte function in arteriogenesis. Interestingly, Peng *et al*. recently reported a male advantage in mouse hindlimb ischaemia where post-ischaemia arteriogenesis was higher in males than in females [26]. This study opens the possibility that an increase in circulating androgen levels may exert therapeutic value for hindlimb ischaemia.

In conclusion, this investigation has demonstrated that AR expression in vascular endothelium is undetectable and makes little contribution to the androgen-dependent regulation of ischaemia-induced angiogenesis. Instead, AR expression in vascular smooth muscle regulates reperfusion of the foot following induction of ischaemia. This suggests that androgen-mediated arteriogenesis makes a significant contribution to recovery from ischaemia. This is the first study to identify the key role of smooth muscle cell AR in regulation of androgen-mediated arteriogenesis, which may in part explain why being male is an advantage in terms of response to ischaemia. These results also have relevance to our understanding of the likely benefit of androgen replacement therapy in patients with ischaemic disease.

## Supporting Information

S1 Fig mRNA expression profile in the gastrocnemius muscles at day21. mRNA expression was normalised to *Gapdh*. * p<0.05 control versus ischaemic, by two way ANOVA. Induction of ischaemia increased transcript number of (A) *Pecam-1*, (B) *Cdh5*, (C) *Vwf*, (D) *Hif1a*, and (E) *Thbs1*, but not (F) *Vegfa*. Selective deletion of vascular ARKO did not alter gene expression in control or ischaemic tissues (n = 6–10).
(TIF)

S2 Fig **AR expression in the ischaemic gastrocnemius muscle in WT (A) and SM/VE-ARKO (B) mice at day 21.** AR expression in the ischaemic gastrocnemius muscle was not affected by vascular ARKO. AR = green; DAPI = Blue.
(TIF)

S3 Fig Vascular ARKO had no effect on muscular injury in ischaemic hindlimb. (A) H&E staining of a cross-section of the gastrocnemius muscle demonstrated intra-muscular adipocytes (arrows) replacing the damaged muscle fibres in the ischaemic hindlimb. (B) Oil red O staining confirmed the presence of lipid-filled adipocytes (arrows) in the ischaemic muscles. (C) The percentage area of intra-muscular adipocytes was quantified using Image-pro plus 7.0. No statistical differences were detected by one way ANOVA (n = 7–10).
(TIF)

S1 Methods (DOCX)

S1 Table qPCR primers used in this study. (TIF)
