# Supplementary figures and images for: Influence of Androgen Receptor in Vascular Cells on Reperfusion following Hindlimb Ischaemia
Source: PLoS One. 2016 May 9;11(5):e0154987. doi: 10.1371/journal.pone.0154987 (PMC4861284; doi:10.1371/journal.pone.0154987)

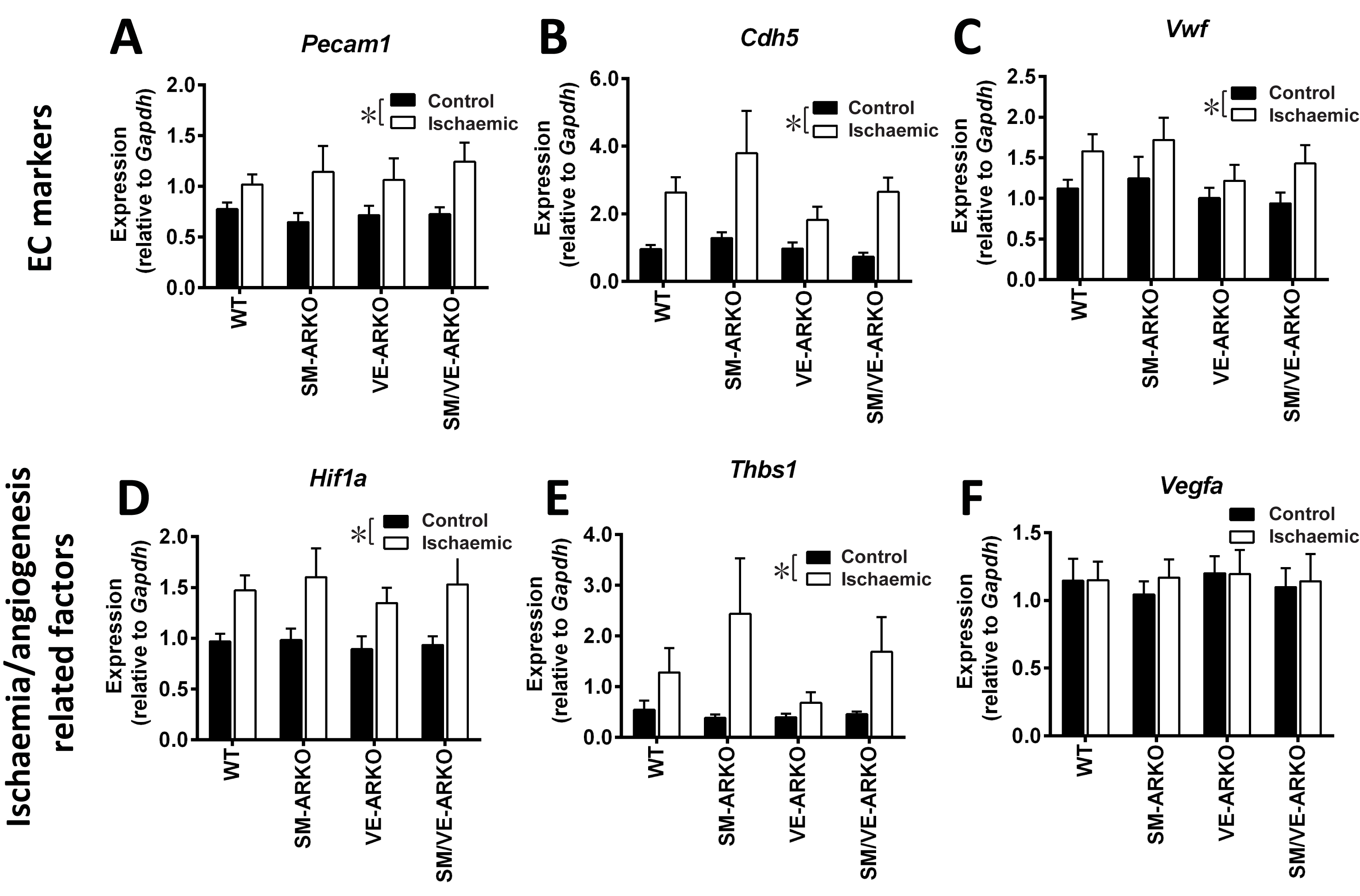

Supplement: S1 Fig — mRNA expression was normalised to Gapdh. * p<0.05 control versus ischaemic, by two way ANOVA. Induction of ischaemia increased transcript number of (A) Pecam-1, (B) Cdh5, (C) Vwf, (D) Hif1a, and (E) Thbs1, but not (F) Vegfa. Selective deletion of vascular ARKO did not alter gene expression in control or ischaemic tissues (n = 6–10). (TIF) [file pone.0154987.s001.tif]

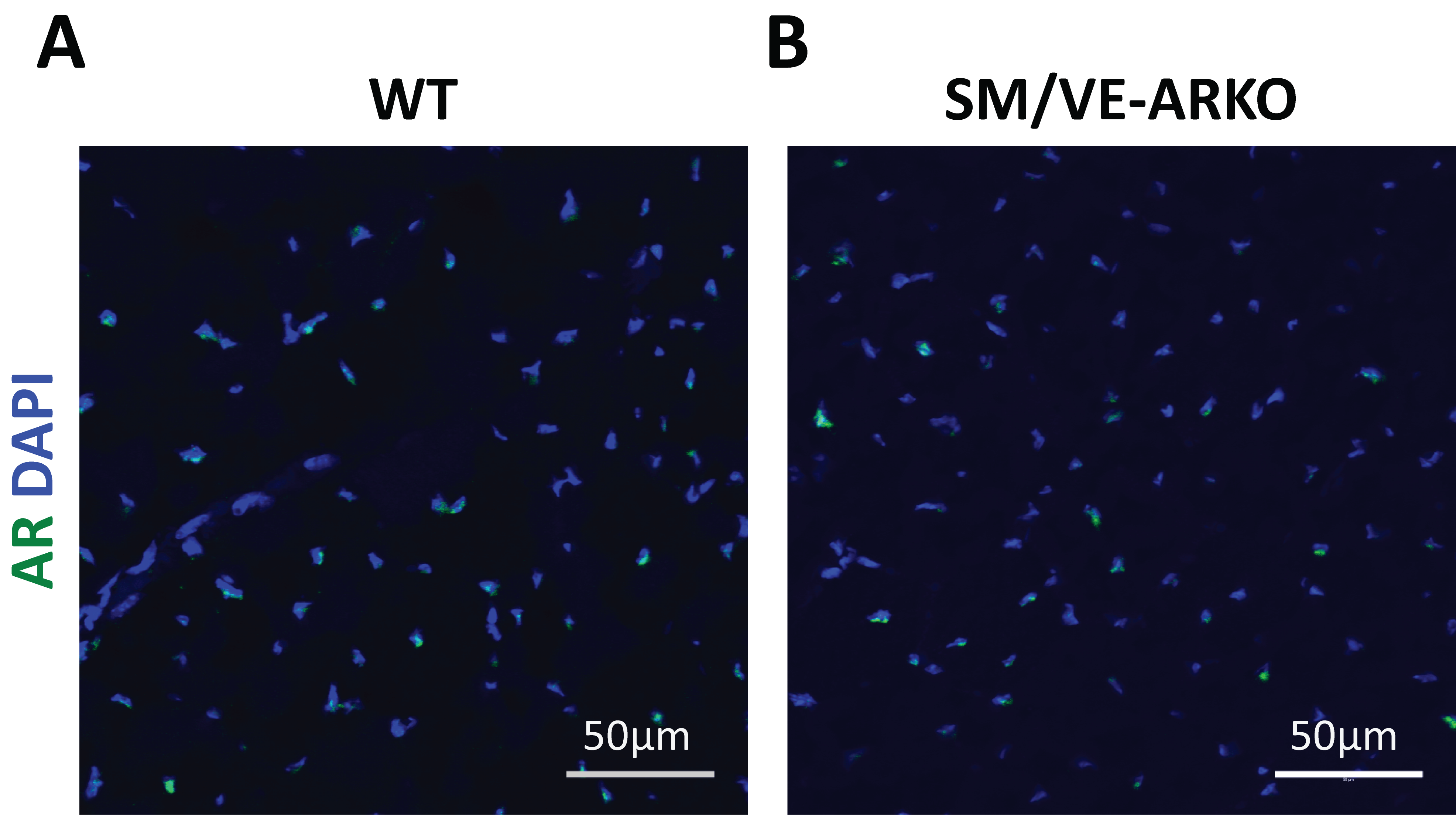

Supplement: S2 Fig — AR expression in the ischaemic gastrocnemius muscle in WT (A) and SM/VE-ARKO (B) mice at day 21. AR expression in the ischaemic gastrocnemius muscle was not affected by vascular ARKO. AR = green; DAPI = Blue. (TIF) [file pone.0154987.s002.tif]

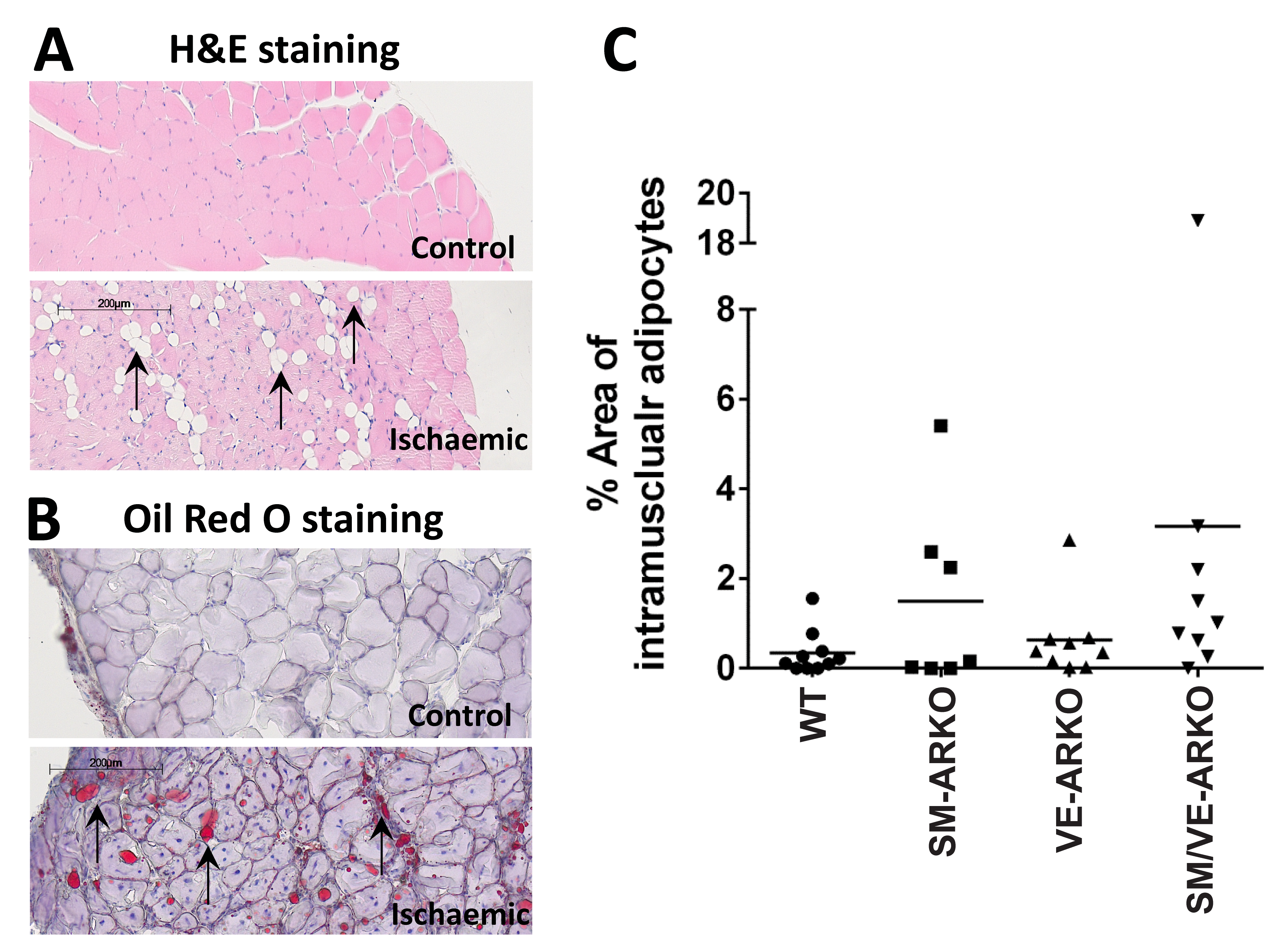

Supplement: S3 Fig — (A) H&E staining of a cross-section of the gastrocnemius muscle demonstrated intra-muscular adipocytes (arrows) replacing the damaged muscle fibres in the ischaemic hindlimb. (B) Oil red O staining confirmed the presence of lipid-filled adipocytes (arrows) in the ischaemic muscles. (C) The percentage area of intra-muscular adipocytes was quantified using Image-pro plus 7.0. No statistical differences were detected by one way ANOVA (n = 7–10). (TIF) [file pone.0154987.s003.tif]

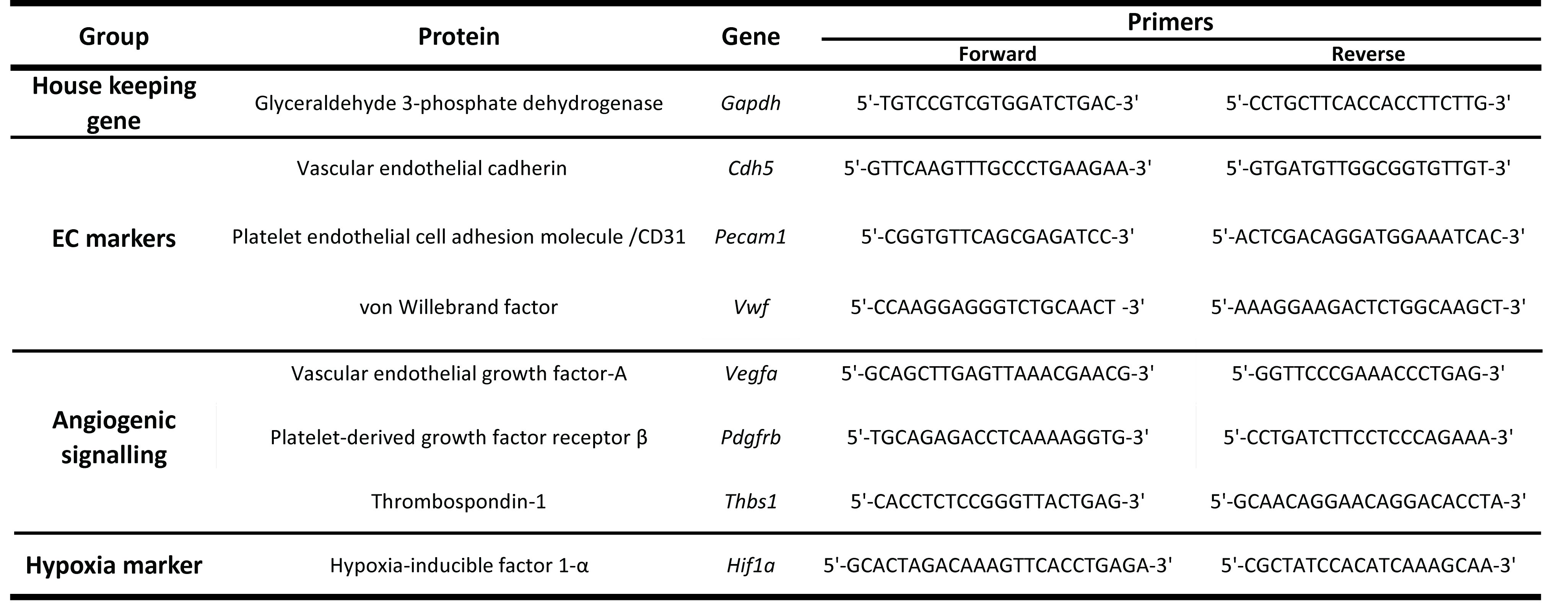

Supplement: S1 Table — (TIF) [file pone.0154987.s005.tif]
