# Supplementary material for: Influence of Androgen Receptor in Vascular Cells on Reperfusion following Hindlimb Ischaemia
Source: PLoS One. 2016 May 9;11(5):e0154987. doi: 10.1371/journal.pone.0154987 (PMC4861284; doi:10.1371/journal.pone.0154987)
Supplement: S1 Methods — (DOCX) [file pone.0154987.s004.docx]

**S1 Methods**

**Quantitative PCR**

RNA was isolated from frozen gastrocnemius muscle from both ischaemic and control legs of each mouse. 40-100mg muscle tissue were homogenised with 500µl QIAzol Lysis Reagent (QIAGEN) using a TissueLyser (QIAGEN). RNA was then extracted into chloroform (Sigma-Aldrich) and further purified using an RNeasy Mini Kit (QIAGEN). RNA concentration and quality were measured using a NanoDrop 1000 Spectrophotometer (Thermo Scientific). RNA samples were stored at -80°C. RNA samples were reverse transcribed to cDNA using a SuperScript VILO cDNA Synthesis Kit (Life Technologies). Quantitative PCR was performed on the Light Cycler 480 system (Roche Diagnostics) for the genes listed (S1 Table). The expression level of each gene was obtained from standard curves of each target gene prepared from serial dilutions of pooled cDNA, and then normalised to *Gapdh*. *Gapdh* expression was similar across groups with no statistical difference detected.

**Oil red staining**

Cryosections from the frozen gastrocnemius muscle were used for oil red O staining to identify lipid-loading adipocytes. Images were analysed using Image-pro plus 7.0 (MediaCybernetics, UK).
